# Characteristics and Mechanisms of Simultaneous Quinoline and Ammonium Nitrogen Removal by a Robust Bacterium *Pseudomonas stutzeri* H3

**DOI:** 10.3390/microorganisms13030687

**Published:** 2025-03-19

**Authors:** Jie Hu, Bing Xu, Jiabao Yan, Guozhi Fan

**Affiliations:** 1 School of Chemical and Environmental Engineering, Wuhan Polytechnic University, Wuhan 430023, China; fgzcch@whpu.edu.cn; 2 Hubei Province Key Laboratory of Coal Conversion and New Carbon Materials, School of Chemistry and Chemical Engineering, Wuhan University of Science and Technology, Wuhan 430081, China; yanmener@wust.edu.cn

**Keywords:** simultaneous nitrogen removal, organic nitrogen, inorganic nitrogen, nitrogen metabolic pathway, whole genome sequencing

## Abstract

The discharge of organic and inorganic nitrogenous pollutants in wastewater leads to eutrophication and disrupts the ecological balance. Therefore, the pressing need for an effective treatment method has become increasingly evident. A robust bacterium *Pseudomonas stutzeri* H3 capable of simultaneous organic and inorganic nitrogen removal was isolated from the activated sludge in the coking wastewater treatment system. The optimal conditions for the simultaneous removal of ammonium nitrogen and quinoline were as follows: C/N ratio of 15–20, initial pH of 7–8, culture temperature of 30 °C, and shaking speed of 150–300 rpm. At 200 mg/L ammonium nitrogen and 100 mg/L quinoline, strain H3 achieved above 90% of removal efficiency, exhibiting excellent simultaneous nitrogen removal capabilities. The outstanding nitrogen removal efficiencies in the presence of quinoline and different inorganic nitrogen sources further confirmed the simultaneous organic and inorganic nitrogen removal capability of strain H3. The whole genome sequencing and nitrogen metabolic intermediates determination of strain H3 were performed to elucidate the gene function annotations, nitrogen removal function genes, and nitrogen metabolic pathways. The findings provide a promising pathway to treat the organic and inorganic nitrogenous pollutants in wastewater.

## 1. Introduction

With the acceleration of industrialization and the expansion of agriculture, plenty of nitrogenous pollutants are discharged into aquatic environments [1,2,3,4], which have become global environmental problems. Nitrogenous pollutants in wastewater are typically classified into two major categories: organic nitrogen and inorganic nitrogen. The organic nitrogenous pollutants predominantly encompass proteins, organic bases, and nitrogenous heterocyclic compounds [5,6,7,8,9]. In contrast, the inorganic nitrogenous pollutants are composed of ammonium nitrogen (NH_4_^+^−N), nitrite nitrogen (NO_2_^−^−N), and nitrate nitrogen (NO_3_^−^−N) [10,11,12]. The discharge of nitrogenous pollutants will not only lead to eutrophication, affecting water quality and ecological balance, but also threaten the health of humans and aquatic animals [13,14,15,16]. Therefore, removing nitrogenous pollutants in wastewater is crucial for the stable discharge of wastewater and the protection of the aquatic ecological environment.

Quinoline is a typical toxic and carcinogenic nitrogenous heterocyclic pollutant, which mainly originates from various industrial processes, such as coking, refining, pharmaceutical manufacturing, and dyeing [4,17,18,19]. Owing to the extreme toxicity and recalcitrant degradability of quinoline, effectively treating quinoline in wastewater has become an intractable problem. Biological nitrogen removal predominantly relies on the metabolic activities of microorganisms to transform nitrogenous pollutants into harmless substances [7,20,21]. In comparison with physicochemical nitrogen removal technology, biological nitrogen removal is characterized by broad applicability, cost-effectiveness, and operational simplicity [14,22,23,24]. Recently, researchers have been exploring efficient biological nitrogen removal processes to remove quinoline from wastewater [17,25,26,27]. For instance, a membrane aerated biofilm reactor (MABR) was constructed to remove quinoline from wastewater under aerobic conditions. More than 80% of quinoline was effectively removed at a quinoline concentration ranging from 50 to 250 mg/L. Metagenomic analysis indicated that aerobic quinoline-degrading bacteria such as *Rhodococcus*, *Pseudomonas*, and *Comamonas* were highly enriched in the MABR biofilm, potentially enhancing the degradation of quinoline in wastewater [25].

The industrial process-discharged wastewater not only contains quinoline but also a significant quantity of inorganic nitrogenous pollutants, such as NH_4_^+^−N and NO_3_^−^−N [2,5,28,29]. Biological nitrogen removal is widely utilized for the elimination of inorganic nitrogenous pollutants present in wastewater [30,31,32,33]. At present, the widely studied technologies for removing inorganic nitrogenous pollutants include shortcut nitrification and denitrification [34,35], anaerobic ammonium oxidation [30,36,37], heterotrophic nitrification and aerobic denitrification [10,11,38]. Heterotrophic nitrification and aerobic denitrification are mainly carried out by some novel nitrogen-removing bacteria in the coexistence of dissolved oxygen and organic carbon sources, thereby effectively removing NH_4_^+^−N and NO_3_^−^−N simultaneously [39,40,41]. Very recently, a novel *Klebsiella* sp. TSH15 was isolated and demonstrated the efficient NH_4_^+^−N and NO_3_^−^−N removal capability. The whole genome analysis indicated that strain TSH15 contained the assimilatory/dissimilatory nitrate reduction and ammonia assimilation functional genes [29].

Previous research efforts are concentrated on the removal of either organic or inorganic nitrogenous pollutants alone [7,14,42], while there are few reports regarding the simultaneous removal of both organic and inorganic nitrogen. The research on the simultaneous biological removal of NH_4_^+^−N and quinoline facilitates the solution of the current water pollution problem and promotes the protection of the ecological environment. In addition, organic nitrogenous pollutants in wastewater are usually converted into inorganic nitrogen, which requires removal by other microorganisms [3,14,16,25]. The main challenge of the simultaneous nitrogen removal process is to identify microorganisms capable of efficiently biodegrading organic and inorganic nitrogenous pollutants and to explore the simultaneous nitrogen removal characteristics and mechanism.

*Pseudomonas* sp. is a common bacterium in wastewater treatment and has excellent nitrogen removal capabilities [4,11,21,38,40]. Wang et al., isolated a quinoline biodegradation bacterium *Pseudomonas citronellolis* PY1, which could effectively remove quinoline in the presence of heavy metal ions Zn^2+^ and Cd^2+^ [21]. An aerobic denitrifying bacterium *Pseudomonas hunanensis* DC-2 was isolated and could remove above 98% of NH_4_^+^−N and 88% of NO_3_^—^N [40].

In this work, a robust bacterium *Pseudomonas stutzeri* H3 capable of simultaneous organic and inorganic nitrogen removal was isolated from the coking wastewater treatment plant (CWTP). Single-factor experiments were performed to optimize NH_4_^+^−N and quinoline removal conditions. The excellent simultaneous NH_4_^+^−N and quinoline removal capabilities of strain H3 were ascertained by comparing with other nitrogen removal bacteria such as *Halomonas* sp. and *Acinetobacter* sp. The nitrogen removal performances for quinoline and different inorganic nitrogen sources were investigated, further confirming the capability of strain H3 to remove both organic and inorganic nitrogen simultaneously. Furthermore, the whole genome sequencing analysis and nitrogen metabolic intermediates determination of strain H3 was performed to elucidate the gene functional annotations and nitrogen metabolic pathways. This study provided new insight into the simultaneous organic and inorganic nitrogen removal by *Pseudomonas stutzeri* H3.

## 2. Materials and Methods

### 2.1. Isolation and Identification

The activated sludge samples taken from the CWTP (Wuhan, China) were utilized to isolate the efficient degrading bacteria for simultaneous removal of NH_4_^+^−N and quinoline. The simultaneous nitrogen removal capabilities of the isolated strain were determined in a mixed nitrogen source (MNS) medium, and its compositions are 8.0 g/L Na_2_HPO_4_·12H_2_O, 1.5 g/L KH_2_PO_4_, 1.0 g/L (NH_4_)_2_SO_4_, 0.1 g/L MgSO_4_·7H_2_O, 0.01 g/L FeSO_4_·7H_2_O, 15.34 g/L sodium succinate hexahydrate, 0.1 g/L quinoline, 2 mL trace element solution. Strain H3 with excellent simultaneous NH_4_^+^−N and quinoline removal efficiency was selected for comprehensive assays. The colony morphology of strain H3 was observed by dilution and plate coating method. The molecular biological method such as 16S rRNA sequencing analysis was utilized to accurately identify the genus of strain H3. Specifically, the genomic DNA of the strain H3 was extracted via standard DNA extraction kit. Polymerase chain reaction (PCR) was used to amplify the 16S rRNA of strain H3. The PCR-amplified product was sequenced by TSINGKE Biotechnology Co., Ltd. (Wuhan, China). Subsequently, the 16S rRNA were uploaded to the GenBank database and aligned with those of other bacteria via online BLAST (http://blast.ncbi.nlm.nih.gov/Blast.cgi, accessed on 26 January 2025). A phylogenetic tree was created using MEGA 7.0. The microscopic morphology of strain H3 was observed by SEM at 3.0 kV (Regulus 8100, Hitachi, Chiyoda, Tokyo).

### 2.2. Effect of Culture Conditions on Nitrogen Removal

The impacts of C/N ratio, initial pH, culture temperature, and shaking speed on NH_4_^+^−N and quinoline removal performance were investigated. Three milliliters of bacterial suspensions was transferred into MNS medium with initial NH_4_^+^−N and quinoline concentrations of 200 and 100 mg/L, and then cultured for 72 h. To investigate the effect of different culture conditions on NH_4_^+^−N and quinoline removal performance, the C/N ratio was regulated to 5, 10, 15, and 20 by changing the amount of additional carbon source of sodium succinate; the initial pH was adjusted to 6, 7, 8, and 9; the culture temperature was regulated at 10, 20, 30, and 40 °C; the shaking speed was varied at 50, 100, 150, and 200 rpm. NH_4_^+^−N and quinoline concentrations were determined at intervals.

### 2.3. Assessment of Simultaneous Nitrogen Removal Capability

To assess the simultaneous NH_4_^+^−N and quinoline removal capability of strain H3, the bacterial suspensions (3 mL) were inoculated into MNS medium. Both NH_4_^+^−N and quinoline were independently set in the range of 50–400 mg/L. NH_4_^+^−N and quinoline removal performances by strain H3 were determined at different concentrations. In addition, *Pseudomonas stutzeri* H3 and other nitrogen removal bacteria *Halomonas* sp. and *Acinetobacter* sp. were cultivated in single NH_4_^+^−N, single quinoline, and mixed NH_4_^+^−N and quinoline media for 72 h. *Halomonas* sp. and *Acinetobacter* sp. were isolated from the aerobic sludge taken from pharmaceutical and coking wastewater treatment systems, respectively. The bacterial growth and NH_4_^+^−N/quinoline removal performance of different bacteria were measured at intervals.

### 2.4. Removal Performance of Strain H3 for Quinoline and Different Inorganic Nitrogen

To assess the nitrogen removal capability of strain H3 in the presence of quinoline and different inorganic nitrogen, the bacterial suspensions were transferred into sterilized media that contained diverse inorganic nitrogen sources (single NH_4_^+^−N, NO_2_^−^−N, NO_3_^−^−N, and mixed NH_4_^+^−N + NO_2_^−^−N + NO_3_^−^−N) and 100 mg/L quinoline. The initial inorganic nitrogen concentration was adjusted to 200 mg/L. The bacterial growth and various nitrogen concentrations were measured at intervals, respectively.

### 2.5. Whole Genome Sequencing and Gene Function Annotations of Strain H3

To elucidate the nitrogen metabolic pathway and conduct an analysis of the gene function annotations of strain H3, whole-genome sequencing was performed on the Illumina Hiseq platform at Majorbio (Shanghai, China). The obtained genome sequences were assembled using SOAPdenovo 2.04 and GapCloser 1.12. The whole genome sequences were submitted and compared with the GO, KEGG, and COG databases to obtain the gene function annotations. The coding DNA sequences associated with nitrogen metabolism were annotated using the KEGG database to identify the nitrogen metabolic pathways present in strain H3.

### 2.6. Determination of Quinoline Metabolic Intermediates

The metabolic intermediates of quinoline degraded by strain H3 were analyzed by GC-MS (Agilent, 7890A GC, 5975C MS, Santa Clara, CA, USA). The bacterial suspensions were transferred into the sterile media that contained 100 mg/L of quinoline. Samples cultivated for 24, 48, and 72 h were collected and centrifuged at 5000 g. The metabolic intermediates of quinoline in the supernatant were extracted by ethyl acetate. The extracts were concentrated by vacuum rotary evaporation. The GC-MS equipped with the HP-5MS capillary column was used to determine the metabolic intermediates of quinoline. The column temperature control programs were initial temperature of 40 °C for 2 min, rose to 300 °C with a heating rate of 7 °C/min, and kept at 300 °C for 10 min.

### 2.7. Analytical Methods

The bacterial growth (OD_600_) was estimated by determining the absorbance of bacterial suspension at the wavelength of 600 nm. The concentrations of NH_4_^+^−N, NO_2_^−^−N, NO_3_^−^−N, and TN were measured through the standard methods [43]. The concentration of quinoline was determined by spectrophotometry at the maximum absorbance wavelength of 313 nm. All experiments were conducted in triplicates and the results were expressed as means ± standard deviation.

## 3. Results and Discussion

### 3.1. Identification of the Isolated Strain H3

**Figure 1:**
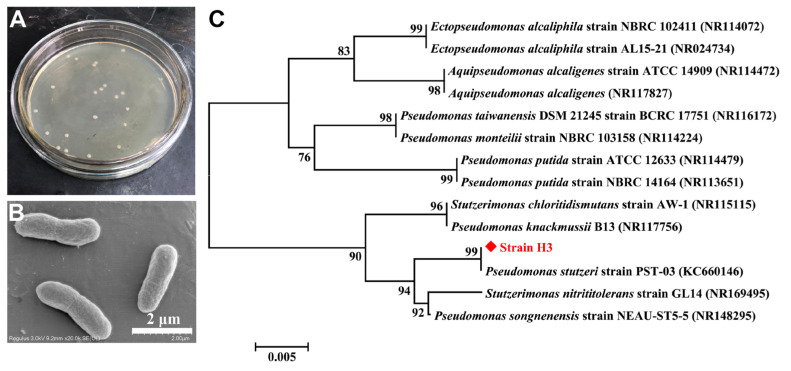
(**A**) The colony morphology of strain H3. (**B**) SEM image of strain H3. (**C**) The phylogenetic tree of strain H3 created by neighbor-joining method with Bootstrap values of 1000 replications.

In this work, ten distinct robust bacteria with simultaneous NH_4_^+^−N and quinoline removal capabilities were isolated from the activated sludge collected from the CWTP. Among these isolated bacteria, strain H3 exhibited the optimal NH_4_^+^−N and quinoline removal performance, achieving a removal efficiency above 90% for both NH_4_^+^−N and quinoline. In contrast, the removal efficiencies of other 9 strains for NH_4_^+^−N and quinoline ranged from 46% to 83%. Therefore, strain H3 was selected for further investigation. The colony of strain H3 was beige, rounded with a diameter around 1 mm, and had a smooth and slightly raised surface (Figure 1A). Moreover, SEM image showed that strain H3 presented as short rods with the size of (0.5 − 0.8) × (2.0 − 3.0) μm (Figure 1B).

After PCR amplification and amplified product sequencing, the 16S rRNA sequences (1434 bp) of strain H3 were obtained and submitted to GenBank database (GenBank ID: PQ899244). The homology analysis by BLAST showed that strain H3 exhibited a high similarity to *Pseudomonas* sp. (above 99% similarity). The phylogenetic tree of strain H3 was created by neighbor-joining method and further demonstrated that strain H3 was affiliated to *Pseudomonas stutzeri* (Figure 1C). At present, *Pseudomonas* sp. widely exists in the wastewater treatment process [44,45,46], but few have been reported to possess the simultaneous NH_4_^+^−N and quinoline removal capabilities.

### 3.2. Optimization of Nitrogen Removal Conditions

**Figure 2:**
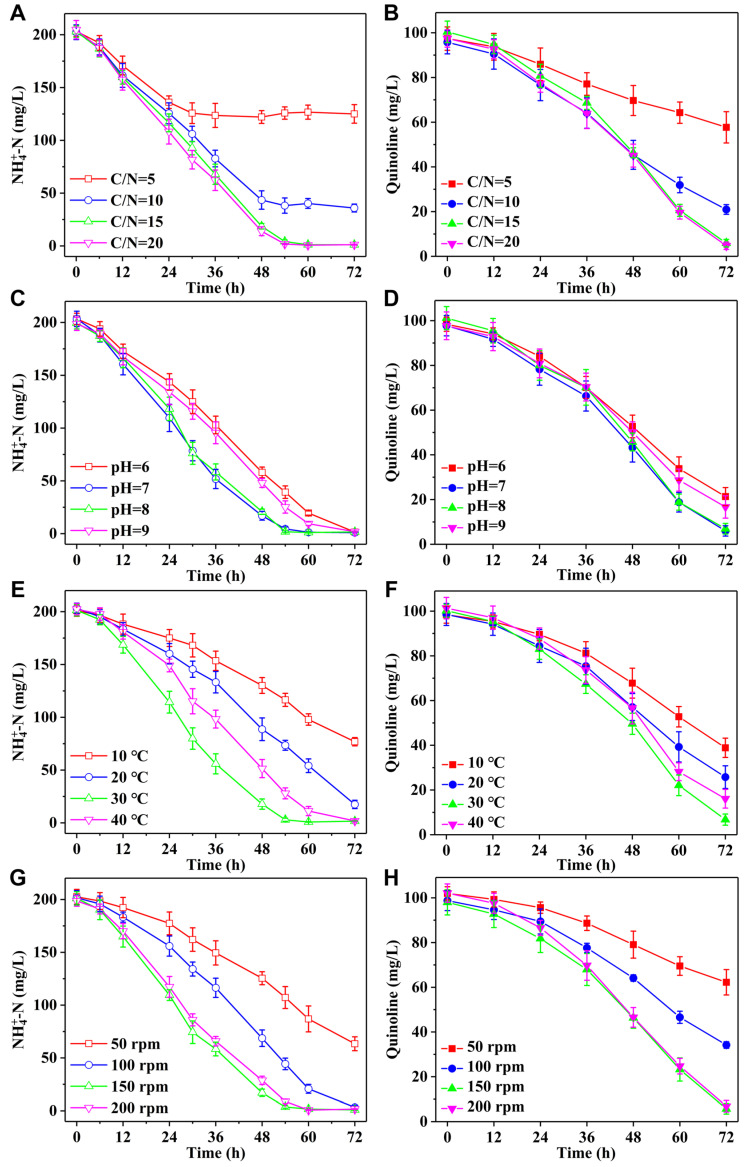
The effects of (**A**,**B**) C/N ratio, (**C**,**D**) initial pH, (**E**,**F**) culture temperature, (**G**,**H**) shaking speed on NH_4_^+^−N and quinoline removal performance of strain H3.

To evaluate the influence of different culture conditions on NH_4_^+^−N and quinoline removal performance of strain H3, single-factor experiments were conducted. Figure 2A,B show the influence of C/N ratio on NH_4_^+^−N and quinoline removal capability. At C/N ratio of 5–20, the removal efficiencies of NH_4_^+^−N and quinoline were gradually enhanced with the increase of C/N ratio. Specifically, when the C/N ratio was 5, the removal efficiencies of NH_4_^+^−N and quinoline were merely 38.5% and 40.8% after strain H3 was cultivated for 72 h. With C/N ratio regulated to 10, the removal efficiencies of NH_4_^+^−N and quinoline were significantly improved to 82.3% and 78.0%. As C/N ratio rose to 15 and 20, the removal efficiencies of strain H3 for NH_4_^+^−N and quinoline were comparable, both reaching above 95%. In the initial pH range of 6–9, strain H3 exhibited excellent NH_4_^+^−N removal properties (Figure 2C). When strain H3 was cultured for 72 h, NH_4_^+^−N removal efficiency was >99%. The removal performances of strain H3 for quinoline varied at different initial pH values (Figure 2D). When strain H3 was cultivated for 72 h, the removal efficiency of quinoline was above 90% at the initial pH of 7–8, which was significantly higher than that of initial pH 6 (78.4%) and 9 (83.1%).

Culture temperature mainly affects the enzyme activity in bacteria, thereby influencing the metabolic activities of bacteria [47,48,49]. As shown in Figure 2E,F, at the culture temperature of 10–40 °C, the removal performances of NH_4_^+^−N and quinoline by strain H3 were significantly different. At the culture temperature of 10 °C, the removal efficiencies of NH_4_^+^−N and quinoline by strain H3 were approximately 60% after cultivation for 72 h. After cultivation at 20 °C for 72 h, NH_4_^+^−N and quinoline removal efficiencies were improved to 91.4% and 74.0%. The removal performances of NH_4_^+^−N were nearly identical after cultivation at 30 and 40 °C for 72 h, but the removal performances of quinoline were obviously different. Specifically, the removal efficiencies of quinoline were 93.3% and 84.3% at 30 and 40 °C. Shaking speed primarily affects the dissolved oxygen (OD) content. Generally, as the shaking speed accelerates, the OD content increases accordingly. As shown in Figure 2G,H, strain H3 exhibited more efficient NH_4_^+^−N and quinoline removal at the shaking speed of 150–200 rpm.

### 3.3. Simultaneous Nitrogen Removal Capability of Strain H3

**Figure 3:**
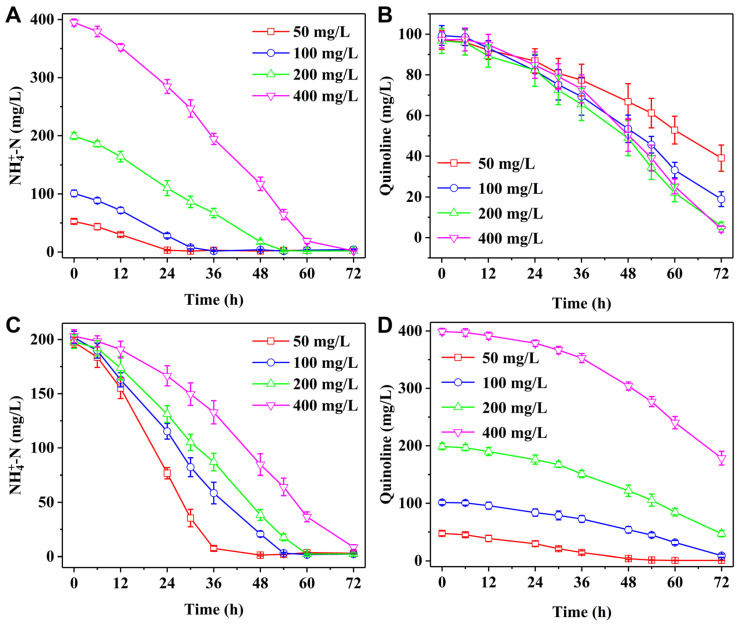
The removal performance of (**A**) NH_4_^+^−N and (**B**) quinoline by strain H3 at the initial concentrations of 100 mg/L quinoline and 50–400 mg/L NH_4_^+^−N. The removal performance of (**C**) NH_4_^+^−N and (**D**) quinoline by strain H3 at the initial concentrations of 200 mg/L NH_4_^+^−N and 50–400 mg/L quinoline.

The growth and quinoline removal performance of strain H3 were studied when only quinoline was used as nitrogen and carbon source. When the quinoline concentration was less than 500 mg/L, strain H3 grew well and could remove above 50% of quinoline after cultivation for 120 h. However, when the quinoline concentration exceeded 1000 mg/L, strain H3 could not grow and remove quinoline. It might be that high concentration quinoline inhibited the growth of strain H3. The simultaneous nitrogen removal capability of strain H3 at different NH_4_^+^−N and quinoline concentrations is shown in Figure 3. At the initial concentrations of 100 mg/L quinoline and 50–400 mg/L NH_4_^+^−N, NH_4_^+^−N was effectively removed by strain H3 after cultivation for 72 h, with a removal efficiency over 95% (Figure 3A). The removal efficiencies of quinoline were enhanced with the increase of initial NH_4_^+^−N at 50–200 mg/L (Figure 3B). However, when NH_4_^+^−N was further raised to 400 mg/L, the removal performance of quinoline was equivalent to that at the NH_4_^+^−N of 200 mg/L. In addition, at the initial concentrations of 200 mg/L NH_4_^+^−N and 50–400 mg/L quinoline, the removal rates of NH_4_^+^−N gradually declined as the quinoline concentration increased (Figure 3C). The removal efficiencies of quinoline by strain H3 decreased from 98% to 55% as the increase of quinoline concentration from 50 to 400 mg/L after cultivation for 72 h (Figure 3D).

**Figure 4:**
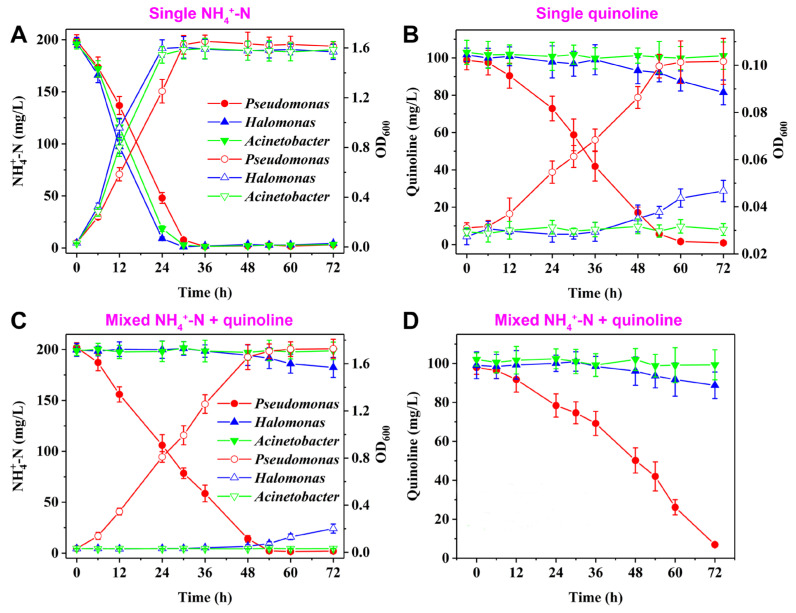
The bacterial growth and nitrogen removal performance of various nitrogen removal bacteria with (**A**), (**B**) quinoline as single nitrogen source, (**C**,**D**) NH_4_^+^−N and quinoline as mixed nitrogen sources. The empty symbols correspond to OD_600_ and the solid symbols correspond to nitrogen removal performance.

When *Pseudomonas stutzeri* H3 and other nitrogen removal bacteria *Halomonas* sp. and *Acinetobacter* sp. were utilized to remove single NH_4_^+^−N, single quinoline, and mixed NH_4_^+^−N and quinoline, the bacterial growth and nitrogen removal performances of different bacteria were shown in Figure 4. With NH_4_^+^−N as single nitrogen source, the removal efficiency of NH_4_^+^−N by *Halomonas* sp. and *Acinetobacter* sp. was slightly higher than that by *Pseudomonas* sp. (Figure 4A). When quinoline was used as single nitrogen source, the bacterial growth and nitrogen removal performance of *Halomonas* sp. and *Acinetobacter* sp. were significantly inhibited, while *Pseudomonas* sp. exhibited excellent bacterial growth and remarkable quinoline removal capability (Figure 4B). When *Pseudomonas stutzeri* H3 was cultivated for 72 h, quinoline removal efficiency was above 95%. When NH_4_^+^−N and quinoline served as mixed nitrogen sources, the removal performances of NH_4_^+^−N/quinoline were different from those of single NH_4_^+^−N and single quinoline (Figure 4C,D). NH_4_^+^−N and quinoline could not be effectively removed by *Halomonas* sp. and *Acinetobacter* sp. in the presence of mixed nitrogen sources. However, when *Pseudomonas stutzeri* H3 was cultivated for 72 h, more than 90% of NH_4_^+^−N and quinoline was effectively removed. The result indicates that strain H3 exhibited excellent simultaneous NH_4_^+^−N and quinoline removal performance.

### 3.4. Simultaneous Removal Performance of Quinolines and Different Inorganic Nitrogen

**Figure 5:**
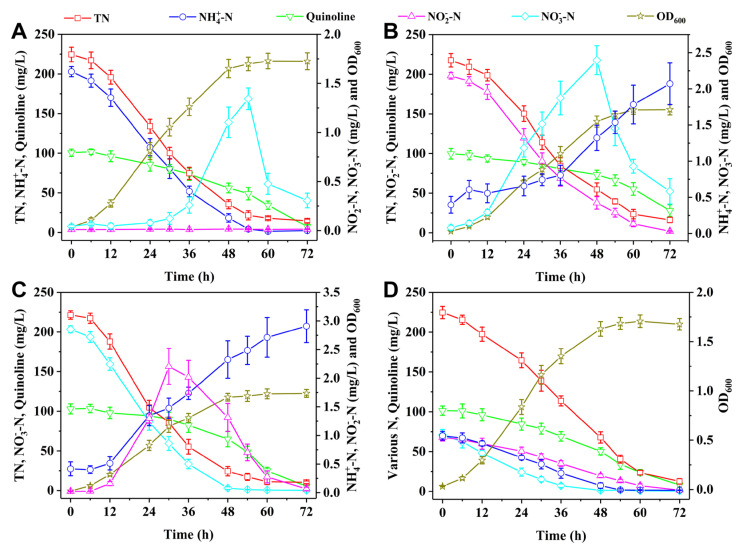
Simultaneous organic and inorganic nitrogen removal performance of strain H3 with (**A**) NH_4_^+^−N + quinoline, (**B**) NO_2_^−^−N + quinoline, (**C**) NO_3_^−^−N + quinoline, and (**D**) NH_4_^+^−N + NO_2_^−^−N + NO_3_^−^−N + quinoline as mixed nitrogen sources.

The bacterial growth and nitrogen removal performance of strain H3 in the presence of quinoline and different inorganic nitrogen sources were shown in Figure 5. When NH_4_^+^−N and quinoline were utilized as mixed nitrogen sources, NH_4_^+^−N and quinoline were simultaneously removed by strain H3 (Figure 5A). More than 98% of NH_4_^+^−N and 92% of quinoline were effectively removed by strain H3 after cultivation for 72 h. The accumulation of NO_3_^−^−N reached a maximum of 1.3 mg/L after cultivation for 54 h, while NO_2_^−^−N remained undetected throughout the culture process. Figure 5B shows the nitrogen removal performance when NO_2_^−^−N and quinoline serve as mixed nitrogen sources. Strain H3 achieved removal efficiencies of 99% for NO_2_^−^−N and 72% for quinoline after cultivation for 72 h. It can be found that the removal performance of quinoline was inferior to that of NH_4_^+^−N and quinoline as mixed nitrogen sources. NO_3_^−^−N reached a maximum accumulation of 2.4 mg/L after cultivation for 48 h and a small quantity of NH_4_^+^−N was detected in the later stage of cultivation.

When NO_3_^−^−N and quinoline were utilized as mixed nitrogen sources, quinoline removal performance was equivalent to that of NH_4_^+^−N and quinoline as mixed nitrogen sources (Figure 5C). Around 95% of quinoline was effectively removed by strain H3 after cultivation for 72 h. The accumulation of NO_2_^−^−N reached a maximum of 2.2 mg/L after cultivation for 30 h. Figure 5D shows that NH_4_^+^−N, NO_2_^−^−N, NO_3_^−^−N, and quinoline used as mixed nitrogen sources can be removed simultaneously by strain H3. The nitrogen removal rates decreased in the order of NO_3_^−^−N > NH_4_^+^−N > NO_2_^−^−N > quinoline. When strain H3 was cultivated for 72 h, the removal efficiencies of NH_4_^+^−N, quinoline, NO_2_^−^−N, NO_3_^−^−N, and TN all exceeded 90%, indicating that strain H3 exhibits outstanding performance in simultaneously removing organic and inorganic nitrogen. *Pseudomonas stutzeri* H3 was isolated from the activated sludge, and its growth and survival are likely to be affected by the specific environmental conditions. In addition, the presence of other microorganisms in the application environment may influence the pathogenic potential of strain H3. Strain H3 may also pose a pathogenic threat to other animals, plants and humans when it enters the environment along with the wastewater discharge. When strain H3 was applied in the actual wastewater treatment system, it is necessary to detect the presence and possible spread of strain H3. In case of any signs of potential risk, mitigation strategies such as the use of biocides or the implementation of additional treatment steps can be considered.

### 3.5. Whole Genome Sequencing and Gene Function Annotations

The whole genome was sequenced and assembled to investigate the genomic characteristics of strain H3. The draft genome of strain H3 was 4,612,156 bp with an average GC content of 64.16% (Figure S1). The whole genome was assembled into 45 scaffolds with N_50_ coverage of 480929 bp. The number of coding genes was 4392, including 68 tRNA genes and 3 rRNA genes. In addition, the number of genes annotated by the databases of GO, KEGG, and COG was 2573, 3373, and 3673 respectively. This variation in numbers implies that different databases have diverse focuses and coverage in gene annotation, which can provide comprehensive insights into the gene functions of strain H3.

**Figure 6:**
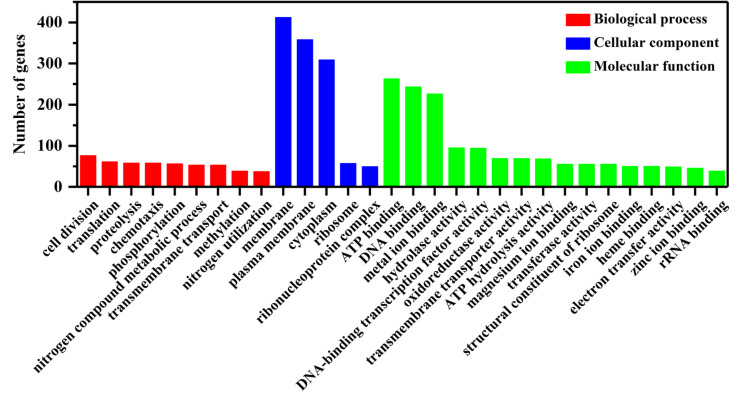
The gene function annotations based on GO database.

Figure 6 and Figure S2 show the gene function annotations of strain H3 by the databases of GO, COG, and KEGG, which are crucial for comprehensive understanding the biological characteristics and metabolic functions of strain H3. The number of gene function annotations by the GO database was 2573, including 1464 in the biological process (BP), 1311 in the cellular component (CC), and 2050 in the molecular function (MF). The top 30 gene function annotations were shown in Figure 6, which contained 9 in the BP category, 5 in the CC category, and 16 in the MF category. The gene function annotations in the BP category related to nitrogen metabolism included 53 in the nitrogen compound metabolic process and 37 in the nitrogen utilization. The gene function annotations based on the KEGG database were initially classified into 6 categories, including cellular processes, metabolism, environmental information processing, genetic information processing, organismal systems, and human diseases (Figure S2A). The number of gene function annotations related to metabolism was the largest. Specifically, carbohydrate metabolism (281) and amino acid metabolism (260) were the primary metabolic activities. In Figure S2B, the gene function annotations based on the COG database are presented and these annotations are divided into 24 categories. It was observed that the numbers of gene function annotations for amino acid transport and metabolism (336) and signal transduction mechanisms (349) were the largest among all the categories.

### 3.6. Nitrogen Metabolic Pathways and Mechanisms

**Figure 7:**
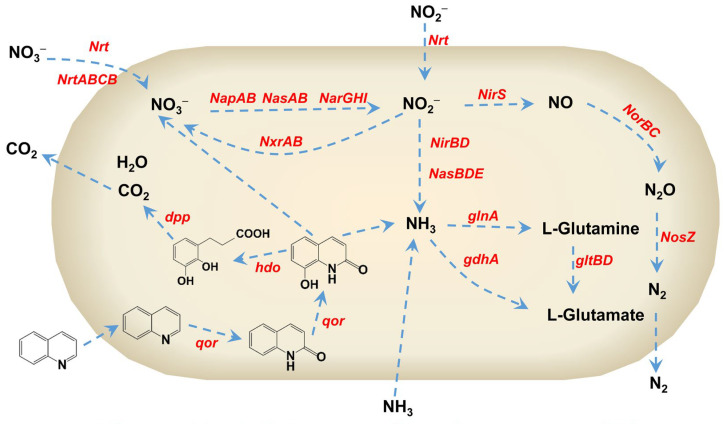
The possible nitrogen metabolic pathway and corresponding nitrogen removal function genes present in strain H3.

The gene function annotations of strain H3 for the nitrogen metabolic pathway based on the KEGG database were shown in Figure 7. Approximately 44 genes related to nitrogen metabolic pathways were annotated, including *NarH*, *NarG*, *NasD*, *NapA*, *NirS*, *NorB*, *NosZ*, etc. The identified nitrogen metabolic pathways included dissimilatory/assimilatory nitrate reduction, denitrification, and ammonia assimilation. The *NarGHI*, *NapAB*, and *NirBD* genes were present in strain H3 and involved in the dissimilatory nitrate reduction. The *NasAB* and *NasBDE* genes were mainly responsible for the assimilatory nitrate reduction. The complete denitrification pathways and associated nitrogen removal functional genes including *NapAB*, *NarGHI*, *NirS*, *NorBC*, and *NosZ* were present in strain H3. Among these nitrogen removal functional genes, *NapAB* and *NarGHI* were responsible for reducing NO_3_^−^−N to NO_2_^−^−N under aerobic and anoxic conditions, respectively [50,51,52]. NH_4_^+^−N was converted into L-Glutamine and L-Glutamate through glutamine synthetase and glutamate synthase encoded by *glnA* and *gltBD* genes. In addition, NH_4_^+^−N could also be converted into L-Glutamate via glutamate dehydrogenase encoded by the *gdhA* gene.

The metabolic intermediates of quinoline degraded by strain H3 were determined by GC-MS analysis. The function genes involved in the degradation of quinoline include *qor*, *hdo*, and *dpp*, etc. The *qor* gene is responsible for encoding quinoline 2,8–dioxygenase (*Qor*), which can oxide quinoline to 2-hydroxyquinoline and further to 2,8-dihydroxyquinoline. The *hdo* gene can encode 2,8-dihydroxyquinoline dioxygenase, which is responsible for cleaving the pyridine ring of 2,8-dihydroxyquinoline to generate 2,3-dihydroxyphenylpropionic acid and inorganic nitrogen. The *dpp* gene clusters encode enzymes that are responsible for breaking down 2,3-dihydroxyphenylpropionic acid to CO_2_ and H_2_O. As shown in Figure 7, the possible metabolic pathway of quinoline degraded by strain H3 was proposed. Quinoline was first converted into 2-hydroxyquinoline by strain H3 under aerobic conditions, which was consistent with the reported quinoline degradation process by other bacteria [3,13,14]. Subsequently, 2-hydroxyquinoline was oxidized to 2,8-dihydroxyquinoline by strain H3. During the biodegradation of quinoline, both NH_4_^+^−N and NO_3_^−^−N were simultaneously detected and rapidly removed. According to the reported work, NH_4_^+^−N and NO_3_^−^−N are often detected during the biodegradation of quinoline [3,5,6,14,16,21,27]. For instance, Tan et al., utilized *Rhodococcus ruber* as the target strain for nitrogen removal. The elemental nitrogen in quinoline was converted into NH_4_^+^−N when quinoline was used as an electron donor [3]. An et al., utilized quinoline-degrading microbial consortium to treat wastewater containing quinoline. NO_3_^−^−N was detected when quinoline was degraded by quinoline-degrading microbial consortium [14]. It is speculated that elemental nitrogen in quinoline was converted into NH_4_^+^−N and NO_3_^−^−N under the action of strain H3. The 2,8-dihydroxyquinoline was further converted into 2,3-dihydroxyphenyl-propionic acid and eventually completely mineralized to CO_2_ and H_2_O. This finding is highly significant for understanding the biodegradation process of quinoline by strain H3, providing a potential solution for the treatment of quinoline in wastewater.

## 4. Conclusions

A simultaneous organic and inorganic nitrogen removal bacterium was isolated and identified as *Pseudomonas stutzeri* H3. Above 90% of both NH_4_^+^−N and quinoline were removed by strain H3 after optimizing the nitrogen removal conditions through single-factor experiments. The excellent simultaneous nitrogen removal capabilities of strain H3 were demonstrated by comparing with other nitrogen removal bacteria *Halomonas* sp. and *Acinetobacter* sp. The outstanding removal performances of quinoline and different inorganic nitrogen sources further confirmed the simultaneous organic and inorganic nitrogen removal capability of strain H3. The gene function annotations, nitrogen removal function genes and nitrogen metabolic pathways of strain H3 were identified by the whole genome sequencing analysis and nitrogen metabolic intermediates determination.

## Data Availability

The original contributions presented in this study are included in the article/Supplementary Materials. Further inquiries can be directed to the corresponding authors.

## Supplementary Materials

The following supporting information can be downloaded at: https://www.mdpi.com/article/10.3390/microorganisms13030687/s1, Figure S1: Circle map of the *Pseudomonas stutzeri* H3 genome; Figure S2: The gene function annotations based on (A) KEGG, (B) COG databases.
