# Supplementary material for: Characteristics and Mechanisms of Simultaneous Quinoline and Ammonium Nitrogen Removal by a Robust Bacterium Pseudomonas stutzeri H3
Source: Microorganisms. 2025 Mar 19;13(3):687. doi: 10.3390/microorganisms13030687 (PMC11945285; doi:10.3390/microorganisms13030687)
Supplement: Supplementary file 1 [file microorganisms-13-00687-s001.zip › microorganisms-3514955-supplementary.pdf]

Supplementary Information

Characteristics and Mechanisms of Simultaneous Quinoline and Ammonium Nitrogen Removal by a Robust Bacterium *Pseudomonas stutzeri* H3

Jie Hu [1,*], Bing Xu [1,*], Jiabao Yan [2] and Guozhi Fan [1]

1 School of Chemical and Environmental Engineering, Wuhan Polytechnic University, Wuhan 430023, China; fgzcch@whpu.edu.cn (G.F.)

2 Hubei Province Key Laboratory of Coal Conversion and New Carbon Materials, School of Chemistry and Chemical Engineering, Wuhan University of Science and Technology, Wuhan 430081, China; yanmener@wust.edu.cn

* Correspondence: hujie9231@whpu.edu.cn (J.H.); xubing200806@163.com (B.X.)

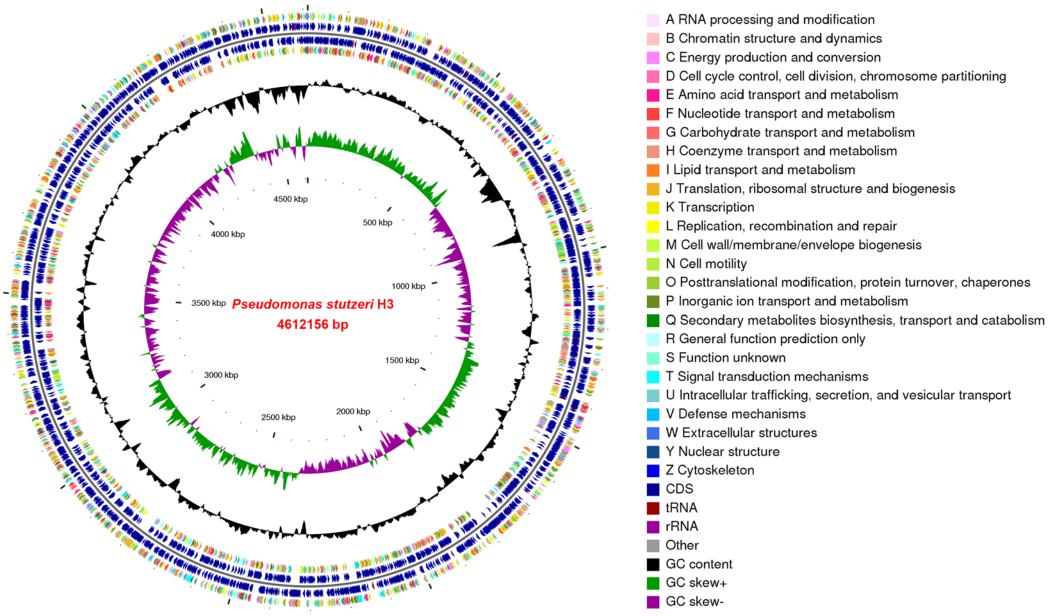

Figure S1. Circle map of the *Pseudomonas stutzeri* H3 genome.

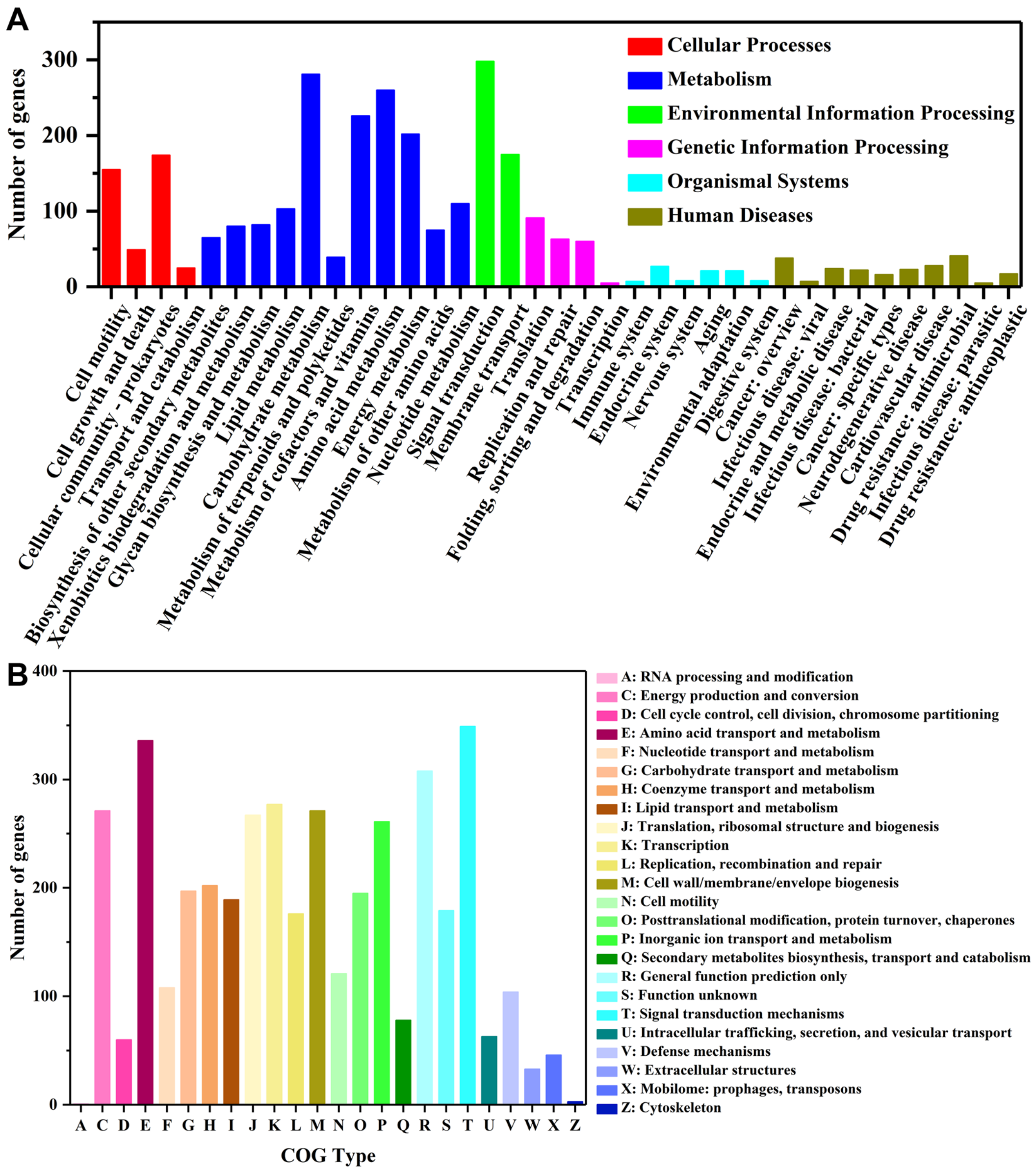

Figure S2. The gene function annotations based on (A) KEGG, (B) COG databases.
